# The Relationship Between Ambulatory Arterial Stiffness Index and Incident Atrial Fibrillation

**DOI:** 10.1002/clc.70007

**Published:** 2024-08-26

**Authors:** Christopher Boos

**Affiliations:** ^1^ Faculty of Health and Social Sciences Bournemouth University Bournemouth UK; ^2^ Department of Cardiology University Hospitals Dorset NHS Dorset UK

We would like to thank Dr Candemir and Kızıltunç for their follow‐up letter in response to our manuscript. They have raised several very important questions. Regarding their first question as to whether there was a statistically significant difference in the diagnosis duration between patients who did and did not develop AF? Unfortunately, we did not collect data on the duration of diagnoses for the cardiovascular risk factors studied and hence will not be able to address this question. We agree this would be an interesting area for future research. It is worth noting that, in addition to duration (which can often be difficult to confirm), the severity of an associated AF risk factor, such as left ventricular ejection fraction (LVEF) in patients with heart failure (HF), is also important. In our manuscript, we observed that the patients who went on to develop AF had a significantly lower LVEF, eGFR, and blood pressure dipping than the non‐AF group, suggesting relatively more severe cardiac and renal dysfunction and poorer hypertension control.

In response to their second point, we have repeated our Cox regression analyses with the additional inclusion of background diagnoses of HF and stroke, both of which were noted to be of greater prevalence in patients who developed AF compared to those who did not. In the full multivariable model, a 1‐SD increase in AASI (HR 1.34; 95% CI 1.04–1.72; *p* = 0.21) and HF (HR 3.47; 95% CI 1.80–6.68; *p* < 0.001) was significantly associated with newly diagnosed AF, along with a history of previous AF, diastolic blood pressure (DBP), but not stroke and hypertension. Repeating the analysis using categorical AASI (above vs. ≤ median), the result was very similar; however, AASI was just above the significance cut‐off (HR 1.65; 95% CI 0.99–2.74; *p* = 0.053).

On their final point regarding additional adjustment for beta‐blocker (BB) use and other medications, repeating the full multivariable analysis with the additional adjustment for background BB use, as well as a 1‐SD increase in AASI, HF, stroke, DBP, sex, previous AF, and hypertension, showed that the independent predictors of new AF were again male, sex, previous AF, lower DBP, HF, AASI (HR 1.36; 95% CI 1.06–1.75; *p* = 0.017), but not previous stroke, hypertension, and BB use. Repeating this analysis using categorical AASI (above vs. ≤ median) rather than continuous AASI revealed comparable results (AASI HR 1.69; 95% CI 1.02–2.81; *p* = 0.043).

Even further analyses with adjustment for the use of calcium channel blockers, ACE inhibitors/angiotensin II receptor blockers, statin use, and mineral corticoid antagonists, in addition to the factors above, revealed equivalent results for both continuous and categorical AASI.

In summary, the results of this manuscript have shown that AASI is a robust and independent predictor of new‐onset AF in a cohort of adults investigated or managed for hypertension.

## Conflicts of Interest

The author declares no conflicts of interest.

## Data Availability

The data relating to this manuscript will be considered on a case‐by‐case basis.

